# Hidden but Potentially Stressed: A Non-Invasive Technique to Quantify Fecal Glucocorticoid Levels in a Fossorial Amphisbaenian Reptile

**DOI:** 10.3390/ani13010109

**Published:** 2022-12-27

**Authors:** José Martín, Isabel Barja, Gonzalo Rodríguez-Ruiz, Pablo Recio, José Javier Cuervo

**Affiliations:** 1 Departamento de Ecología Evolutiva, Museo Nacional de Ciencias Naturales, C/José Gutiérrez Abascal 2, 28006 Madrid, Spain; 2 Etho-Physiology Group, Unit of Zoology, Department of Biology, Faculty of Sciences, Autonomous University of Madrid, 28049 Madrid, Spain; 3 Centro de Investigación en Biodiversidad y Cambio Global (CIBC-UAM), Autonomous University of Madrid, C/Darwin 2, 28049 Madrid, Spain

**Keywords:** amphisbaenian, endocrine activity, enzyme immunoassay, fecal corticosterone metabolites, non-invasive monitoring, reptiles, *Trogonophis*

## Abstract

**Simple Summary:**

Soil alterations may negatively affect the health of animals living inside these soils, but these negative effects are often unexplored and remain “hidden” underground. This study examines the validity of a non-invasive technique to quantify glucocorticoid levels of the amphisbaenian *Trogonophis wiegmanni*, a fossorial burrowing reptile. Quantification of corticosterone metabolites was made from fresh fecal samples using an enzyme immunoassay kit. An experimental external supplementation of corticosterone to a group of amphisbaenians was detected in their feces as an increase in their fecal glucocorticoid metabolite levels, confirming that this treatment can be used to induce physiological increases of this hormone in these animals. We also quantified baseline fecal glucocorticoid metabolite levels in a field population of this amphisbaenian using this technique. Results showed that although there were no differences between sexes, sizes, or seasons, there was a high interindividual variation, which may allow using this measurement and technique to examine in detail the environmental causes that may produce this variation.

**Abstract:**

To understand wildlife responses to the changing environment, it is useful to examine their physiological responses and particularly their endocrine status. Here, we validated an enzyme immunoassay (EIA) to non-invasively quantify fecal corticosterone metabolites (FCM) in the fossorial amphisbaenian reptile *Trogonophis wiegmanni* from North Africa. We supplemented animals assigned to the treatment group with corticosterone dissolved in oil applied non-invasively on the skin for several days, while control groups received the oil-alone solution. Fresh feces were collected at the end of the supplementation period, and FCM levels were quantified by an EIA. Basal FCM levels were similar for both treatments and increased at the end of the test, but FCM increased significantly more in corticosterone-treated animals. A further examination of FCM levels in a wild population of this amphisbaenian did not find overall sexual, size or seasonal differences but showed a high range of variation among individuals. This suggests that different uncontrolled intrinsic or local environmental variables might increase the circulating glucocorticoid levels of different individuals. Our results confirmed the suitability of EIA for analyzing physiological changes in FCM in this amphisbaenian species. This technique may be useful for understanding and remediating the little-explored potential stressors of the soil environment that may negatively affect the health state of fossorial reptiles.

## 1. Introduction

Understanding the causal mechanisms underlying conservation problems, such as the health state and decline of populations derived from global change, is needed to manage and confront these threats adequately. In this context, it is important to study the physiological responses of organisms in free-living populations to understand how animals are affected by perturbations of the environment and whether they can maintain stability (homeostasis), decreasing negative impacts on fitness, despite rapidly changing environmental conditions [1,2,3]. Some physiological indicators and techniques are being used more frequently nowadays because they can allow a rapid assessment of the causes of conservation problems, as well as suggest conservation actions and assess their effectiveness [3]. Physiological stress is one of the health indicators that have been considered for this purpose, as it may reflect the responses of animals to changes in the environment [2,4]. Glucocorticoids (GC) levels have been used as physiological indicators in several studies of many vertebrate species, e.g., [5,6]. Particularly in wildlife studies, a non-invasive analytical technique has emerged that consists of quantifying GC levels through the residual metabolites of GCs found in fresh feces [7,8,9]. This measure correlates with measures of free GCs in plasma [10] and avoids harmful invasive sampling techniques.

Alterations expected from the global change could have particularly strong negative consequences for the soil and underground environment [11,12]. In addition to climate change, which directly affects soil temperature and humidity, there could be impacts derived from, for example, soil pollution by fertilizers or heavy metals and soil degradation (e.g., compaction, salinization, or erosion) [12]. These soil disturbances will strongly and directly affect the health state of soil biodiversity, e.g., [12,13,14,15,16]. However, these negative impacts have been scarcely studied and may remain “hidden” underground, resulting in a bad but unnoticed conservation state of populations. This is because the importance of soil biodiversity as a key factor in regulating the functioning of terrestrial ecosystems is often not appreciated [12,17], and little concern about the conservation of fossorial animals is evident [18].

Amphisbaenians are among the more notable fossorial reptiles, but they have been understudied because they spend their life buried underground, being a little conspicuous and difficult to study [19,20]. Amphisbaenians are morphologically and functionally adapted to a strict fossorial life, showing reduced vision and limb loss [19,20,21], which affect many aspects of their ecology, e.g., [22,23,24]. Fossorial reptiles may face different conservation threats than epigeal ones [18]. For example, amphisbaenians must be especially affected by small-scale local soil alterations, as they spend all their lives underground, and their mobility is restricted to small home ranges [15,25,26]. However, only a few studies have examined the potential impact of soil disturbances on fossorial reptiles [17,18], and the consequences for the conservation of their populations are, therefore, not well understood. Analyzing the physiological health state and particularly the variations in GC levels of free-living populations of fossorial animals in response to soil alterations and other potential stressors may contribute to their management and conservation.

Here, we validated a technique to quantify GC levels of the Northwest African checkboard amphisbaenian *Trogonophis wiegmanni*. The main purpose was to optimize and validate the suitability of an enzyme immunoassay (EIA) for non-invasively quantifying fecal corticosterone metabolites (FCM). We first made a controlled experiment using an external supplementation of corticosterone and examined whether supplemented individuals increased FCM levels more than control ones. Then, we used the validated assay for a preliminary exploration of seasonal, sex, and size-related differences in FCM levels in the field in an island population of this amphisbaenian.

## 2. Materials and Methods

### 2.1. Study Area

**Figure 1:**
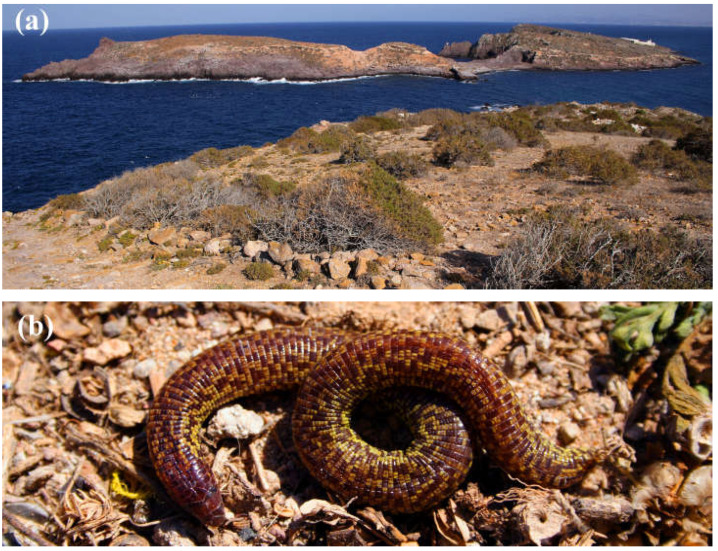
(**a**) Typical habitat of Isabel II Island (Chafarinas Islands) where *T wiegmanni* amphisbaenians were studied, with Rey Island in the background; (**b**) an adult *T wiegmanni* amphisbaenian as it was found under a stone.

We performed fieldwork at the Chafarinas Islands (Spain) (Figure 1a). These islands are located in the Mediterranean Sea (35°11′ N, 02°25′ W), close to the North African coast (Ras el Ma, Morocco). The archipelago has three small islands: Isabel II (15.1 ha), Congreso (25.6 ha), and Rey Francisco (13.9 ha), which have restricted access. Soils are poorly developed and immature [27]. The vegetation, dominated by woody bushes (*Salsola*, *Suaeda*, *Lycium*, and *Atriplex*), is adapted to soil salinity and drought conditions resulting from an arid and warm Mediterranean climate [28].

### 2.2. Study Animals and Sampling Procedures

The amphisbaenian *T. wiegmanni* (Figure 1b) is found in North Africa, from Morocco to northeast Tunisia [29]. Like in other amphisbaenians, the knowledge of its ecology is limited, but this is likely one of the amphisbaenian species with more information on several aspects of its ecology and behavior, e.g., [15,16,25,26,30,31,32,33,34]. The conservation state of this species was considered by the IUCN as of “Least Concern” [35], but more information is required, and its actual conservation problems are unknown.

We made two field campaigns at the Chafarinas Islands in early autumn (September-October) of 2019 and spring (March) of 2020, spending two weeks in each campaign conducting fieldwork. Every day (between 07:00 and 18:00 h GMT), we lifted rocks to search for amphisbaenians that were found under them, and we captured live animals by hand. Amphisbaenians usually defecated most gastrointestinal content while being handled as an anti-predatory response. Thus, we made use of this behavior commonly exhibited upon handling (convenience sampling) to collect fresh fecal samples, but on some occasions, we also forced the expulsion of feces by gently massaging the bellies. If an individual did not defecate in a few seconds, we released it without taking samples. We stored the feces of each individual in an Eppendorf vial. Samples were maintained cold inside a portable refrigerator during fieldwork and later stored at −20 °C in a freezer.

The total body ‘length’ of amphisbaenians (from the tip of the snout to the tip of the tail) was measured (to the nearest 1 mm) using a metallic ruler. We also used a digital scale to weigh (to the nearest 0.01 g) the body ‘mass’ of animals after extracting their fecal samples to avoid confounding effects of fecal mass on body mass. A ‘body condition index’ was calculated as the residuals of the least squares linear regression between body mass and total length (both log_10_-transformed) (linear regression, *r* = 0.86, *F*_1,177_ = 505.59, *p* < 0.0001). This index is considered a proxy of the health state in many animals because the residuals allow for the separation of the condition effects from those of body size [36,37]. We determined the sex of each individual by examining the presence of hemipenes in the cloacae [30,38]. Thereafter, we released amphisbaenians at their exact point of capture less than 5 min after finding them. However, 24 individuals were maintained temporarily in captivity for a corticosterone supplementation experiment (see below) and released 12 days after capturing with good health and without observing any loss of body condition in any individual.

### 2.3. Biological Validation

This experiment was conducted in March 2020. Before the experiment, 24 adult amphisbaenians of similar body size were captured on Isabel Island and immediately taken to the nearby Chafarinas Biological Field Station, located on the same island less than 100 m from the capture sites. Amphisbaenians were maintained for 24 h before and during the experiment in individual cages (25 × 15 cm) with a thin sand substrate from the study area. During the experiment, the amphisbaenians were fed insect larvae and snails collected in the same area [32]. Water was provided daily with a water spray. Cages were placed in a laboratory room with large open doors so that the ambient temperature and the photoperiod were similar to those of the surrounding habitat where the animals had been captured.

We experimentally increased circulating corticosterone (CORT) levels of amphisbaenians using a non-invasive method. For 12 days, 12 individuals (6 females, 6 males) randomly assigned were daily supplemented with 5 μL of a solution of 2 μg corticosterone (≥92%, C2505, Sigma Aldrich, Saint Louis, MO, USA) dissolved in 6 mL of soybean oil (430005, Sigma-Aldrich) applied with a pipette on the dorsum (CORT treatment), while the other 12 individuals (6 females, 6 males) were supplemented with soybean oil alone (Control treatment). High concentrations of lipids in reptiles’ skin make lipophilic molecules to be quickly absorbed into the bloodstream [39]. This procedure has been proven effective in increasing corticosterone levels in the blood of several species of lizards [39,40,41] and snakes [42]. In order to avoid potential bias due to the time of the day’s influence on metabolism and excretion of GC [43], all animals received the CORT or control treatment at the same time (between 9:00 h and 10:00 h local time). Cages of experimental and control individuals were spatially mixed and maintained under the same conditions. We collected fecal samples (see above) of control and CORT-supplemented amphisbaenians just before the experiment began and the day after (12 h after) the supplementation process had ended. However, at the end of the experiment, we could only collect enough amounts of fecal samples that were useful for analyses from 19 individuals (9 control and 10 experimental).

### 2.4. Measurement of Fecal Corticosterone Metabolites (FCM)

Frozen fecal samples were sent from the field inside portable freezers for analyses at the Ethology and Endocrinology Lab (Madrid Autonomous University) of Madrid, maintaining the cold chain from collection to analyses to avoid the effects of different storage temperatures on GC levels [44,45]. In the lab, all samples were stored frozen at −20 °C for less than one month since collection to analyze to avoid the effects of storage time on FCM levels [7].

To extract FCM in the feces of amphisbaenians, we first pulverized and dried the frozen fecal samples in an oven at 90 °C for 4 h. Then, 0.05 g of each dry sample was placed into an Eppendorf tube, where we added 500 μL of phosphate buffer saline (PBS) and 500 μL of 100% methanol. To homogenize the sample and the solvents, we agitated tubes using first a vortex shaker and later an orbital shaker, where tubes were kept for 16 h. The extract obtained was centrifuged at 2500× *g* rpm for 15 min, and the supernatant was transferred to polyurethane tubes (suitable for hormone preservation) that were kept at −20 °C until being quantified. Less than a week after extraction, we used a commercial EIA kit (D-24145; Demeditec Diagnostics GmbH, Kiel, Germany) for the quantification of FCM in fecal extracts. The quantification of FCM levels (in ng/mL dry excrement) in the fecal extracts was done using a spectrophotometer (Microplate Reader, MR 600; Dynatech Industries Pvt. Ltd., Bangalore, India). We processed fecal samples in random order and made duplicated analyses of the fecal extracts.

We calculated the parallelism, accuracy, and precision tests to validate the EIA tests used. We compared serial dilutions (1:16, 1:8, 1:4, 1:2, 1:1) of pooled fecal extracts with the standard curve provided by the manufacturer to calculate the parallel displacement curves (*R*^2^ = 0.94, *p* > 0.05). The high accuracy of the recovery of corticosterone (above 90%) showed that the fecal extracts did not have compounds that could interfere with the quantification. The precision of the quantification was also high, as showed by the low intra- (9.6%) and inter-assay (11.9%) coefficients of variation for fecal samples. The sensitivity of the assay for corticosterone metabolites was greater than 4.1 ng/mL.

### 2.5. Statistical Analyses of Data

To analyze the effects of the corticosterone supplementation experiment, we used a General Lineal Model (GLM) to estimate whether individual log_10_ transformed FCM levels of amphisbaenians, as the response variable, changed from the beginning to the end of the experiment (‘time’ effect as a repeated measures factor that considers within individual variation), depending on their ‘treatment’ (Control vs. CORT) and ‘sex’ (male vs. female), both as categorical fixed factors and including the interactions between factors in the model.

To test for differences in FCM levels of amphisbaenians observed in the field, we used a GLM to estimate whether individual log_10_ transformed FCM levels, as the response variable, differed depending on the ‘sex’ of the individual or the ‘season’ (spring vs. autumn) when it was sampled (both as fixed factors), and included the log_10_ transformed body ‘length’ as a continuous covariate. We also included all two-way and three-way interactions in the model.

We ensured that residuals of the models fulfilled the assumptions of normality and homoscedasticity (using Shapiro–Wilk’s and Levene’s tests, respectively). Statistical analyses were made with the Statistica 8.0 software (StatSoft Inc., Tulsa, OK, USA).

## 3. Results

### 3.1. Biological Validation

**Figure 2:**
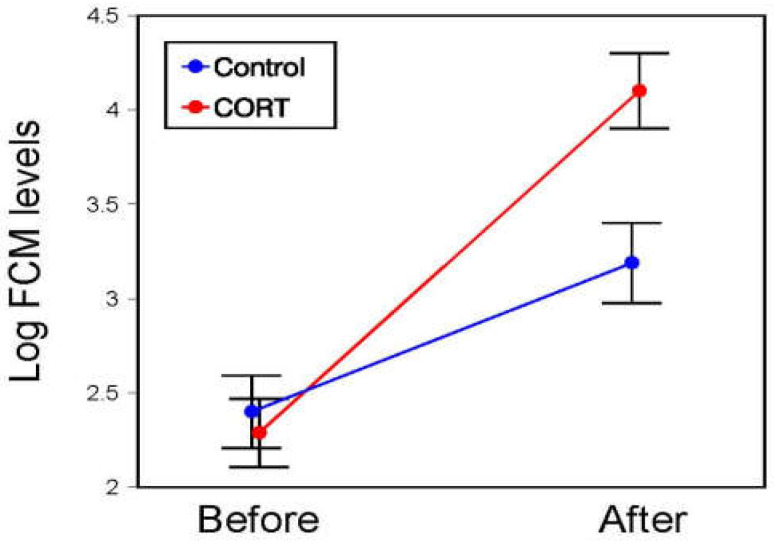
Mean (±SE) levels of fecal corticosterone metabolites (log_10_-transformed FCM; ng/g dry excrement) before and after a corticosterone supplementation experiment of control (blue) and experimental (CORT; red) individual *T. wiegmanni* amphisbaenians.

Individual overall FCM levels of amphisbaenians did not significantly differ between treatments or sexes, but FCM levels increased significantly from the beginning to the end of the experiment (‘time’ effect), and the significant interaction between ‘time’ and ‘treatment’ indicated that CORT treated individuals increased more their FCM levels with time than control individuals (Table 1; Figure 2). Thus, while control and CORT individuals did not significantly differ in their initial FCM levels (Tukey’s test, *p* = 0.98), both groups increased their initial FCM levels with time (Tukey’s tests, Control, *p* = 0.048; CORT, *p* < 0.0002), but at the end of the treatment, CORT individuals had significantly higher FCM levels than control ones (Tukey’s test, *p* = 0.028). Thus, all individuals had increased their FCM levels at the end of the experiment, but while in control individuals, their final raw FCM levels were, on average, 13 times higher than the initial ones. In CORT individuals, final FCM levels were 327 times higher than initial levels. The sex of the amphisbaenian did not significantly affect this effect of the CORT treatment.

### 3.2. Seasonal, Sexual and Size-Related Variation in Baseline Field FCM Levels

**Figure 3:**
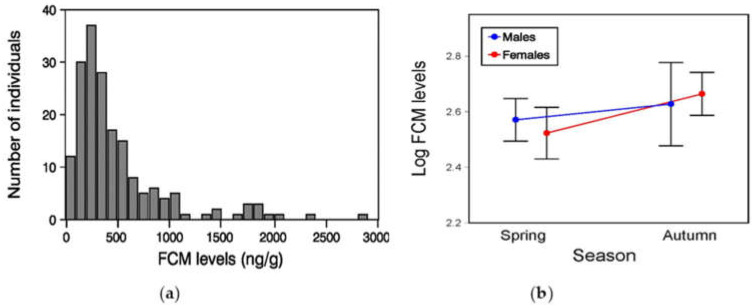
(**a**) Frequency distribution of baseline levels of fecal corticosterone metabolites (FCM; ng/g dry excrement) of individual *T wiegmanni* amphisbaenians; (**b**) Sex and seasonal variation in mean (±SE) log_10_-transformed FCM levels.

The FCM levels of amphisbaenians in the field were on average (±SE) 495 ± 35 ng of FCM/g dry feces, although inter-individual variability was high and, thus, although most individuals (76.4%) had FCM levels between 50 and 600 ng/g, there were 23.6% of individuals that had higher values between 600 and 2850 ng/g (Figure 3a). However, average FCM levels did not significantly differ between ‘seasons’ (GLM, *F*_1,171_ = 2.77, *p* = 0.10) or ‘sexes’ (*F*_1,171_ = 2.62, *p* = 0.11) (Figure 3b) and were not significantly correlated with body ‘length’ (*F*_1,171_ = 0.40, *p* = 0.53). All two-way and three-way interactions were no significant (season × sex: *F*_1,171_ = 1.06, *p* = 0.30; season × length: *F*_1,171_ = 2.70, *p* = 0.10; sex × length: *F*_1,171_ = 2.62, *p* = 0.11; season × sex × length: *F*_1,171_ = 1.04, *p* = 0.31). Furthermore, FCM levels of amphisbaenians were not significantly related to their ‘body condition index’ (Pearson’s correlation, *r* = 0.11, *F*_1,177_ = 2.13, *p* = 0.15).

## 4. Discussion

Inter- and intra-specific variability in the characteristics of the physiological stress responses observed in studies with different animal species [46,47] indicated the importance of optimization and validation of non-invasive quantification of GC levels for each new species where this technique is intended to be used. Our study first confirmed that the experimental corticosterone supplementation increased FCM concentrations in the amphisbaenian *T. wiegmanni*, which supports the suitability of using the corticosterone EIA as a valid method for the non-invasive quantification of FCM concentrations in wild populations of this amphisbaenian. Furthermore, our findings indicated that neither the average baseline FCM concentration nor the effects of the corticosterone supplementation differed between sexes. The results also confirmed the previous finding that the application on the skin of corticosterone dissolved in oil might be used to increase corticosterone levels in reptiles for experimental purposes [39,40,41,42]. This offers the possibility of combining these two procedures (skin corticosterone application and measurement of FCM levels) to design future experimental studies examining the effects of potential stressors in this amphisbaenian.

Interestingly, the application of oil alone (control) also increased FCM in *T. wiegmanni* amphisbaenians, although to a significantly lesser degree than corticosterone. This may suggest that captivity conditions and human handling procedures alone may also act as potential stressors for sensitive individuals. Similar responses to saline controls in ACTH tests have been observed in, for example, some rodents [47,48]. This highlights the need for careful control of housing and handling procedures to minimize the disturbance of animals during the experimental work, not only because of animal welfare considerations [4] but also because the increased GC levels of control animals may confound the results of some experiments examining behavioral or physiological consequences of potential stressors. Alternatively, it is known that adding mineral oil to the skin of lizards can lead to permeabilization of the skin that is followed by a large increase in rates of cutaneous water loss, leading to dehydration, which might also explain part of the increase in the FCM levels, being both groups of animals affected by the oil supplementation. However, it is unlikely that the low amounts of oil used in our experiment caused a significant loss of water, and, moreover, we did not observe any significant difference in the body condition of animals between the start and the end of the supplementation period.

By using this validated FCM assay, we made a preliminary exploration of sex, body size (a partial proxy for age) [30], and seasonal differences in FCM levels in a single wild population of *T. wiegmanni* amphisbaenians. The overall results of a large sample did not show any significant difference in mean FCM values between sexes, body sizes, or seasons. While, for example, during the breeding season, males of other reptiles often show slightly higher levels of GC as a consequence of costly reproductive behaviors [49,50,51], we did not find this effect in this amphisbaenian species. These results might be initially interpreted as a lack of sexual, size, or reproductive effects in this species, but they might rather result from the confounding effects of other uncontrolled environmental or intrinsic variables that may mask the potential basal sexual, age, and seasonal variations, which might otherwise be observed under “ideal” conditions. In fact, we found a wide range of variation in FCM levels in the wild, suggesting that in some individuals, the levels of circulating corticosterone were higher than in others, independently of their sex, size, or season. The observed variability could indicate that individuals may differently perceive stressful stimuli and accordingly undergo different hormonal adjustments to each stressful event or that environmental variation of potential stressors has not been considered. This result prompts future studies examining the uncontrolled environmental or intrinsic causes that may correlate with these interindividual variations in GC levels. For example, a previous study examining the FCM levels in different populations of *T. wiegmanni* showed a correlation between FCM levels and heavy metal concentrations in the soil, with populations inhabiting more contaminated areas showing higher FCM levels [52]. The validation of the FCM measurement test made in the current study supports the idea that these high FCM levels may represent a physiological increase of GCs, likely as a consequence of the disruptive endocrine effects of heavy metals [53].

We did not find a significant relationship between the body condition of amphisbaenians and their FCM levels in the wild. Body condition has been used as a proxy for the health state of reptiles [37,54]. For example, soil salinization and food restrictions due to extended drought conditions negatively affected the body condition index of *T. wiegmanni* [15,55], while contamination by heavy metals did not affect body condition [15], although it did increase FCM levels [52]. This leads to the recommendation to obtain a reliable, complete perspective of the health state of an individual or population. We should combine several physiological measures and indexes and control for the potential confounding effects of several environmental variables.

In many vertebrates, also in reptiles, high levels of GC in blood and feces were considered as a proxy of stress, e.g., [51,56,57,58,59]. Nevertheless, increases in GC per se are not necessarily indicative of stress, as these hormones have complex, interactive effects across many systems [60,61]. Measuring physiological stress responses is important for monitoring populations because chronic high-stress levels have been shown to result in several physiological damages, such as decreased immune responses, and often lead to suppression of reproduction, e.g., [62,63,64,65,66]. Therefore, a population with highly stressed individuals could decline quickly in the future. The assay that we have validated here for measuring FCM levels in *T. wiegmanni* can be useful and important to quickly detect changes in the GC levels of individual amphisbaenians and relate this variation with potential soil disturbances before the entire population is negatively affected. Moreover, this technique is a convenient innocuous sampling that takes advantage of a behavior (expulsion of feces as an anti-predatory behavior) that these animals usually exhibit when captured. In contrast, blood sampling of these small-sized amphisbaenians would not provide enough amount of blood useful for GC analysis without serious damage to animals. Likewise, similar methods and assays could be used for other fossorial animals in the future.

## 5. Conclusions

The health and conservation state of populations of animal species living underground has often been understudied due to the difficulty of finding and sampling these animals. This is important in a changing world scenario, where many threats to soil biodiversity that may occur hidden in the soil could be easily overlooked. The measurement of physiological parameters using simple and non-invasive techniques, such as determining the FCM levels with EIA, can be a strong tool to monitor and manage populations of fossorial animals. Validation and optimization of such methods for each target species are, however, required to ensure the accuracy and reliability of the health estimations in future studies of wild populations [47,67]. The continuous monitoring of the physiological health state of amphisbaenian populations in the field, together with environmental data, may help to understand the negative factors that affect these animals and to predict and take actions to minimize potential future conservation problems derived from global change.

## Figures and Tables

**Table 1:** Results of a GLM testing the effects of a corticosterone supplementation experiment on the fecal corticosterone metabolites (FCM) levels of the amphisbaenian *T. wiegmanni*.

	SS	df	*F*	*p*
Treatment	1.51	1	3.68	0.07
Sex	0.27	1	0.66	0.43
Treatment × Sex	0.13	1	0.33	0.58
Error	6.15	15		
Time	15.93	1	50.19	<0.0001
Time × Treatment	2.47	1	7.78	0.014
Time × Sex	0.02	1	0.06	0.81
Time × Treatment × Sex	0.38	1	1.19	0.29
Error	4.76	15		

## Data Availability

The data presented in this study are openly available in FigShare at https://doi.org/10.6084/m9.figshare.21581463 (accessed on 25 December 2022).
